# At last, mobile health leading to a diagnosis in a young patient with congenital heart disease

**DOI:** 10.1007/s12471-018-1224-z

**Published:** 2019-01-08

**Authors:** M. A. C. Koole, G. A. Somsen, I. I. Tulevski, M. M. Winter, B. J. Bouma, M. J. Schuuring

**Affiliations:** 1 Cardiologie Centra Nederland, Utrecht, The Netherlands; 2 Department of Cardiology, Rode Kruis Ziekenhuis, Beverwijk, The Netherlands; 3 Department of Cardiology, Amsterdam UMC, University of Amsterdam, Amsterdam, The Netherlands; 4 Haga Teaching Hospital, The Hague, The Netherlands

**Fig. 1:**
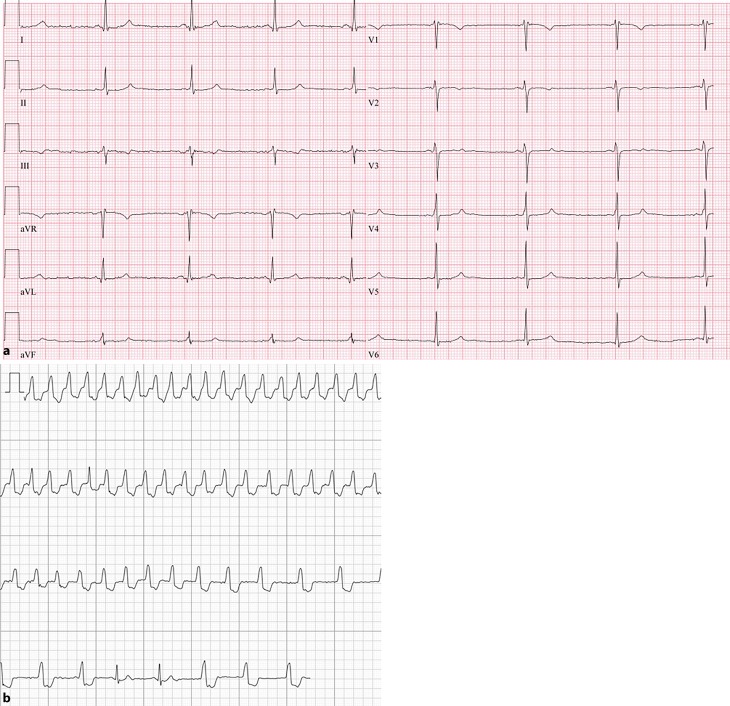
**a** Routine resting electrocardiogram (ECG) of the patient. **b** Recording of atrial fibrillation with intermittent bundle branch block by on-demand single-lead ECG in the mHealth program

A woman with congenital pulmonary valve stenosis treated by balloon valvulotomy had been visiting the outpatient clinic for many years with complaints of palpitations. Several 24-h electrocardiogram (ECG) recordings and resting ECGs (Fig. 1a) showed frequent premature ventricular contractions and two regular wide-complex asymptomatic tachycardias of a maximum of eight beats with a maximal frequency of 120 beats/min. However, these findings could not be related to her complaints.

The patient was included in a novel mHealth telemonitoring program. While the patient was having palpitations, a single-lead ECG was recorded (Fig. 1b). On these additional recordings atrial fibrillation could be diagnosed with intermittent bundle branch block.

There is a broad aetiology of palpitations, and these often occur outside the window of 24-h Holter monitoring. This case is one of the first to illustrate that mHealth programs, including on-demand ECG monitoring, can be of great importance in the diagnosis of arrhythmias.

